# The Reversal Effect and Its Mechanisms of Tetramethylpyrazine on Multidrug Resistance in Human Bladder Cancer

**DOI:** 10.1371/journal.pone.0157759

**Published:** 2016-07-08

**Authors:** Shanshan Wang, Ting Lei, Man Zhang

**Affiliations:** 1 Clinical Laboratory Medicine, Beijing Shijitan Hospital, Capital Medical University, Beijing, China; 2 Beijing Key Laboratory of Urinary Cellular Molecular Diagnostics, Beijing, China; University of Pecs Medical School, HUNGARY

## Abstract

Chemotherapy is an important strategy for the treatment of bladder cancer. However, the main problem limiting the success of chemotherapy is the development of multidrug resistance (MDR). To improve the management of bladder cancer, it is an urgent matter to search for strategies to reverse MDR. We chose three kinds of herbal medicines including ginsenoside Rh2, (-)-Epigallocatechin gallate (EGCG) and Tetramethylpyrazine (TMP) to detect their effects on bladder cancer. Reversal effects of these three herbal medicines for drug resistance in adriamycin (ADM)-resistant Pumc-91 cells (Pumc-91/ADM) were assessed by Cell Counting Kit-8 (CCK-8) cell proliferation assay system. The mechanisms of reversal effect for TMP were explored in Pumc-91/ADM and T24/DDP cells. After Pumc-91/ADM and T24/DDP cells were treated with TMP, cell cycle distribution analysis was performed by flow cytometry. The expression of MRP1, GST, BCL-2, LRP and TOPO-II was evaluated using quantitative real-time polymerase chain reaction (qRT-PCR), immunefluorescence assay and western blot. It was observed that TMP was capable of enhancing the cytotoxicity of anticancer agents on Pumc-91/ADM cells in response to ADM, however Rh2 and EGCG were unable to. The reversal effect of TMP was also demonstrated in T24/DDP cells. Moreover, the treatment with TMP in Pumc-91/ADM and T24/DDP cells led to an increased of G1 phase accompanied with a concomitant decrease of cell numbers in S phase. Compared to the control group, an obvious decrease of MRP1, GST, BCL-2 and an increase of TOPO-II were shown in TMP groups with a dose-dependency in mRNA and protein levels. However, there was no difference on LRP expression between TMP groups and the control group. TMP could effectively reverse MDR of Pumc-91/ADM and T24/DDP cells and its mechanisms might be correlated with the alteration of MRP1, GST, BCL-2 and TOPO-II. TMP might be a potential candidate for reversing drug resistance in bladder cancer chemotherapy.

## Introduction

Globally, bladder cancer is the most common cancer of the genitourinary tract in men [1]. Approximately 70% of cancers are non-muscle invasive tumors with high recurrence, while the remaining 30% are muscle invasive with high risk of death from distant metastases [2]. The transurethral resection of bladder tumor (TURBT) is essential for non-muscle invasive bladder cancer treatment. With regard to low-grade Ta and T1 tumor, intravesical chemotherapy or immunotherapy is necessary. As for muscle-invasive bladder cancer, radical cystectomy and lymph nodes dissection is the standard operation [3]. Systemic chemotherapy is a reasonable alternative after surgery for patients with muscle invasive bladder cancers. Recent studies show that surgery combining with chemotherapy can improve the quality of life and improve survival [4]. However, cancer cells frequently develop an almost uncanny ability to resist the effects of cancer chemotherapeutic agents. Selection of cancer cells with one chemotherapeutic drug usually results in cross-resistance to other drugs with different cellular targets and structures. This phenomenon is known as multidrug resistance (MDR) [5]. The development of multidrug resistance in bladder cancer cells can severely impair the success of cancer systemic chemotherapy [6]. Adriamycin and cisplatin are important drugs used for chemotherapy against bladder cancer. However, due to the development of MDR, the treatment in bladder cancer with adriamycin, cisplatin or other agents often fails. The acquisition of MDR could be mediated via many mechanisms, including the increase in drug efflux, the decrease in drug influx, drug inactivation and alterations in the drug target, modification of cell cycle checkpoints and increased DNA damage repair and defective apoptotic pathways [7–9].

Some MDR proteins involve in the drug resistance of bladder cancer via reducing the intracellular drug concentrations. These proteins can predict poor outcomes after chemotherapy [10–12]. In bladder cancer, the expression of MDR1 mRNA in recurrent and residual tumors after doxorubicin chemotherapy was higher than that in untreated primary tumors [13]. Multidrug resistance protein 1 and lung resistance related protein were overexpressed in locally advanced bladder cancer. MRP1 expression correlated with a higher response and a higher probability of bladder preservation following neoadjuvant chemotherapy [14]. High LRP expression was significantly associated with a worse response to neoadjuvant chemotherapy and a decreased probability of bladder preservation [14].

Many multi-drug resistance modulators have been reported for their contribution to MDR. However because of the present several barriers, they limited therapeutic efficacy in the clinic. One common reason for clinical failure of MDR modulators is their non-specific toxicity to cancer patients. Another obstacle is the unexpected and undesired pharmacokinetic interactions between the modulators and the anti-cancer drugs used for the treatment of patients, which results in reducing doses of anticancer drugs and so inefficient benefit [15]. Therefore, it is an urgency to find novel MDR mediators which can effectively reverse the drug resistance to satisfy the requirement of chemotherapy.

**Fig 1:**
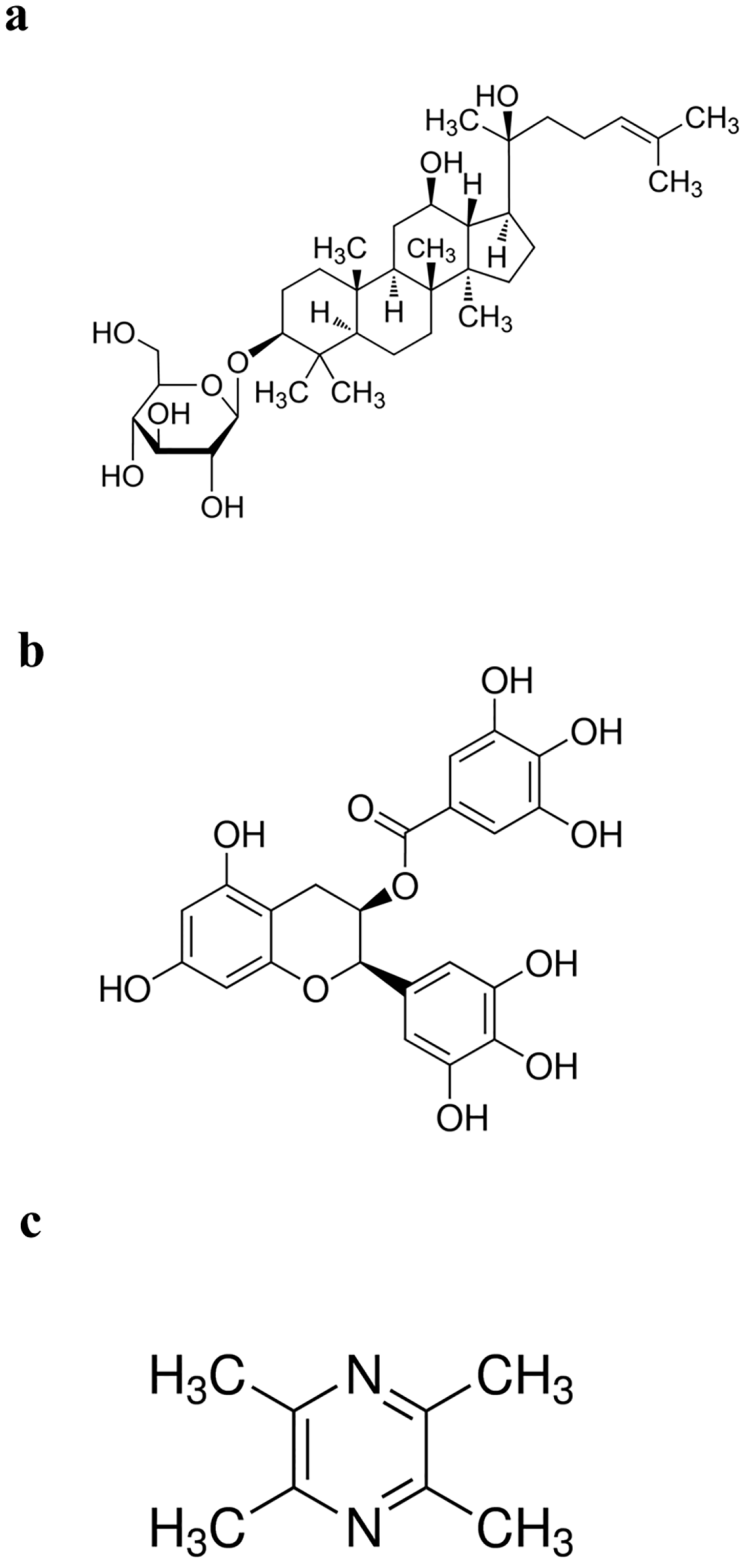
The structure of three kind of herbal medicine. a. Rh2, b. EGCG, c.TMP.

Rh2 (Fig 1a), a ginsenoside with a dammarane skeleton, has been shown to suppress growth and induce apoptosis in vitro in many cancer cells [16–18]. Green tea, derived from the plant Camellia sinensis, is one of the most common beverages consumed worldwide, especially in China. The major catechins in green tea is (-)-epigallocatechin-3-gallate (EGCG) (Fig 1b) which accounts for 50–80% of catechin in green tea. It has been reported that EGCG has inhibitory activity against tumorigenesis [19,20]. Tetramethylpyrazine (Fig 1c) is one of the major bioactive components of Chuanxiong. It was confirmed that TMP was capable of reversing the MDR of BEL-7402/ADM cells in response to ADM [21]. But so far there were no studies on reversing drug resistance of Rh2, EGCG and TMP in bladder cancer chemotherapy.

In this study, the effects of Rh2, EGCG and TMP on cell viability were detected in multidrug resistant human bladder cancer cell line Pumc-91/ADM. The reversal effects of these three herbal medicines were also performed. Moreover, the reversal effect mechanisms of TMP on MDR were studied in Pumc-91/ADM and T24/DDP cells.

## Materials and Methods

### Cell culture

The human bladder cancer cell line Pumc-91 was provided by the Cell Laboratory of Peking Union Medical College Hospital. Pumc-91/ADM was a drug-resistant cell line that was established by stepwise exposure of Pumc-91 cells to escalating concentrations of adriamycin (aladdin, China), ranging from 0.02 μg/ml to 1.00 μg/ml for more than 6 months. The human bladder cancer cell line T24 was provided by Chinese Academy of Sciences Culture Collection. The human drug-resistant bladder cancer cell line T24/DDP was established by stepwise exposure of T24 cells to escalating concentrations of cisplatin (sigma), ranging from 0.01 μg/ml to 0.60 μg/ml for more than 6 months. The drug-resistant characteristics of Pumc-91/ADM and T24/DDP cell line were confirmed by MTT (3-[4, 5-dimethylthiazol-2-yl]-2, 5-diphenyl tetrazolium bromide) assay, reverse transcription polymerase chain reaction (RT-PCR) and flow cytometry in previous studies [22,23]. The parental cell line was cultured in RPMI-1640 medium (Gibco, Invitrogen) which was supplemented with 10% heat-inactivated fetal bovine serum (Dingguo, Biotechnology), while drug-resistant cell lines were cultured in the above-mentioned medium with 18% fetal bovine serum. Cells were incubated at 37°C in a standard 5% CO_2_ cell incubator with a humidified atmosphere of 95% air. All experiments were carried out while cells were in exponential growth phase.

### Cell viability assay

The effects of Rh2, EGCG and TMP on the viability of Pumc-91/ADM cells were measured using the Cell Counting Kit-8 (CCK-8) (dojindo) cell proliferation assay system. Pumc-91/ADM cells were seeded on 96 well plates with a density of 8×10^3^ cells per well. After overnight incubation, cells were incubated with various concentrations of Rh2 (0–100 μM) (Sigma-Aldrich), EGCG (0–200 μM) (Sigma-Aldrich) and TMP (0–32 mM) (Sigma-Aldrich). Rh2 and EGCG were dissolved in dimeth-ylsulfoxide (DMSO) at a final concentration of less than 0.1% and TMP was dissolved in methyl alcohol at a final concentration of less than 0.1%. DMSO and methyl alcohol at a final concentration of less than 0.1% have no effect on cell viability. RPMI-1640 culture medium without cells served as a vehicle control and RPMI-1640 culture medium with cells served as a negative control. The cytotoxicity of these three traditional Chinese medicines was measured via CCK-8 assay. After cells were treated with Rh2, EGCG and TMP for 24h, 48h and 72h, 10μl of CCK-8 solution was added into each well. The absorbance was measured at a wavelength of 450 nm and 655 nm using a microplate reader (Model 680, BioRad Laboratories, Hercules, CA, USA) after cells incubated for 2h at 37°C. The proliferation ratio was calculated using the following equation: proliferation ratio (%) = (OD _the drug treated group_−OD _the control group_) / (OD_the control group_—OD _the vehicle group_). The half maximal inhibitory concentration (IC_50_) was defined as the concentration of the drug that resulted in 50% inhibition of cell growth. IC_50_ was obtained via regression analysis between drug concentrations and the cell inhibition rate using SPSS 17.0 software. All of the experiments were repeated three times.

### Multidrug resistance reversal assay

The reversal effect of Rh2, EGCG and TMP on Pumc-91/ADM cells was further detected with CCK-8. To minimize the effect of Rh2, EGCG and TMP themselves on cell growth, we chose lower concentrations of them in the reversal experiments. Cells were prior to seeding into 96-well plates and then treated with varying concentrations of ADM in the presence of Rh2 (0, 5, 10, 20 μM), EGCG (0, 10, 20, 40μM) and TMP (0, 1, 2, 4 mM) for 48h, respectively. Cells treated without herbal medicine were considered as the control group. Meanwhile, the reversal effect of TMP on T24/DDP cells was further detected with CCK-8. As described above, 10 μl of CCK-8 solution was added into each well. After cells were incubated at 37°C for 2h, the absorbance was recorded at 450 nm and 655 nm wavelength.

The reversal fold (RF) values, as potency parameter, were calculated from dividing IC_50_ of ADM alone by IC_50_ of ADM in combination with Rh2, EGCG or TMP in Pumc-91/ADM cells. RF of TMP in T24/DDP cells was calculated from dividing IC_50_ of DDP alone by IC_50_ of DDP in combination with TMP. The combinational index (CI) was calculated using the formula: CI = (Ea+b)/(Ea+Eb-Ea×Eb) [24]. In the formula, Ea+b represented the inhibition rate of ADM in combination with Rh2, EGCG, TMP or DDP in combination with TMP. Ea represented the individual inhibition rate of Rh2, EGCG or TMP and Eb represented the inhibition rate of ADM or DDP. The nature of the drug interaction was defined as: i) Additive (+) if CI ranged from 0.85 to 1.15; ii) synergism (++) if CI ranged from 1.15 to 2.0; iii) subtraction (-) if CI ranged from 0.85 to 0.55 and iv) antagonism (—) when the CI was <0.55.

### Cell cycle distribution analysis

Pumc-91/ADM and T24/DDP cells were plated in culture dishes at concentrations determined to yield 50–60% confluence within 24h. Cells were then treated with different concentrations of TMP (0, 2 and 4 mM) for 48h. After that, cells were washed twice with cold dulbecco's phosphate-buffered saline (D-PBS) (Invitrogen) and then centrifuged at 140g for 5 min. The pellets were fixed with 70% ethanol in D-PBS and stored at 4°C for 1h. And then, cells were washed with D-PBS and resuspended with propidium iodide solution (0.01 mg/ml) (Sigma-Aldrich) containing RNase (100 μg/ml). After that, cells were incubated in the dark at room temperature for 30 min. The DNA content was then analyzed using the FACSCalibur flow cytometer with CellQuest software (BD Biosciences, San Jose, CA, USA). The data were analyzed using modfit software.

### qRT-PCR

Total RNA was isolated with trizol reagent (Ambition, life technologies, USA) from Pumc-91/ADM and T24/DDP cells which were treated with TMP (0, 2 mM and 4 mM) according to manufacturer’s instructions. cDNA was generated using Oligo d (T) (Dingguo Biotech), dNTP (Genview) under the action of M-MLV reverse transcriptase (Promega). Quantitative real-time PCR was done by using TransStart Top Green qPCR SuperMix (Beijing TransGen Biotech Co., Ltd) with LightCycler 480 Real Time PCR System. The PCR primer sequences used were as follows: GAPDH forward: 5’-TTTGGTATCGTGGAAGGACT-3’ and reverse: 5’-AGTAGAGGCAGGGATGATGT-3’; MRP1 forward: 5’-TTGCCGTCTACGTGACCATT-3’ and reverse: 5’-AGGCGTTTGAGGGAGACACT-3’; LRP forward: 5’-TATGTGCCATCTGCCAAA GT-3’ and reverse: 5’-CATGTAGGTGCTTCCAATCA-3’; GST forward: 5’-TTCCTGTGGCATAATGTGAT-3’ and reverse: 5’-CTGATTCAA AGGCAAATCTC-3’; TOPO-II forward: 5’-AGGCATCGCATCTTGTTTAG-3’ and reverse: 5’ -CTGTCTCCGGTCTTCCATAA-3’; BCL-2 forward: 5’-GACAACATCGCCCTGTGGAT-3’ and reverse: 5’- AGGGCCAAACTGAGCAGAGT-3’. Quantitative real-time PCR were done by using TransStart Top Green qPCR SuperMix (Beijing TransGen Biotech Co., Ltd) with LightCycler 480 Real Time PCR System. GAPDH was used as endogenous reference. Relative gene expression was calculated using the 2-ΔΔCt method [25]. All of the experiments were repeated three times.

### Immunofluorescence

When cultured to 90% confluence, cells were seeded on a glass slide at the density of 1×10^5^ cells/ml and incubated with TMP (0, 2 mM, 4 mM) for 48h. After that, cells were washed three times with phosphate-buffered saline (PBS) (Hangzhou Sijiqing Company) and then fixed with 4% paraformaldehyde (Dingguo Biotechnology) for 15 min at room temperature. Cells were permeabilized in 0.1% Triton X-100/PBS (Sigma-Aldrich) for 10 min and incubated with 10% goat serum for 1h at 37°C. Cells were incubated with primary antibody of mouse monoclonal anti-MRP1 (1:100), rabbit monoclonal anti-LRP (1:100), rabbit polyclonal anti-GST (1:250), rabbit monoclonal anti-BCL-2 (1:100), rabbit polyclonal anti-TOPO-II (1:500), respectively (Abcam) at 37°C for 2h. After being washed with PBS three times, the cells were stained with AlexaFluor 488-conjugated secondary antibody (goat anti-mouse and goat anti-rabbit IgG, Abcam, 1:400) at 37°C in a moist cassette for 1h. The cells were washed with PBS three times and then incubated with Hoechst 33342 at the concentration of 5μg/ml for 10min at room temperature. Cell images were subsequently captured with fluorescence microscopy (Nikon 80i, Japan, ×400).

### Western Blot

Pumc-91/ADM and T24/DDP cells were treated with TMP (0, 4 mM). After incubated for 48h, cells were collected and washed three times with PBS when in exponential growth phase. Protein extracts were prepared using a total protein extraction kit (KeyGen, Nanjing, China) according to the manufacturer's instructions. Protein (80 μg) per sample was separated in 8–12% SDS-PAGE and then was electrotransferred to a PVDF membrane (Bio-Rad, USA) for 1.5h. The membranes were blocked in solution with 5% nonfat milk in PBST for 2h at room temperature. Afterwards, the membranes were incubated with mouse or rabbit primary antibodies (Abcam, USA) against MRP1, LRP, GST, BCL-2 and TOPO-II at dilutions of 1:250, 1:1000, 1:250, 1:100, 1:500, respectively and β-actin (ZhongShan Co., China) at a dilution of 1:500 at 4°C overnight. Then the PVDF membrane was incubated in horseradish peroxidase-conjugated goat anti-mouse IgG antibody or anti-rabbit IgG antibody at a dilution of 1:1000 for 1.5h at room temperature. The bands were detected by enhanced HRP-DAB chromogenic kit. The integrated optical density (IOD) value of every band was analyzed with Image-Pro Plus v 6.0 software (MEDIA CYBERNETICS, USA). All of the experiments were repeated three times.

### Statistical analysis

Statistical analysis was done by the SPSS 17.0 software (SPSS lnc., Chicago, USA). Student’s t test (two-tailed) and analysis of variance (one-way ANOVA) were used to analyze the control group and TMP groups. P value less than 0.01 was considered to indicate significant difference.

## Results

### Growth inhibitory effect of three kinds of herbal medicines in Pumc-91/ADM cells

**Fig 2:**
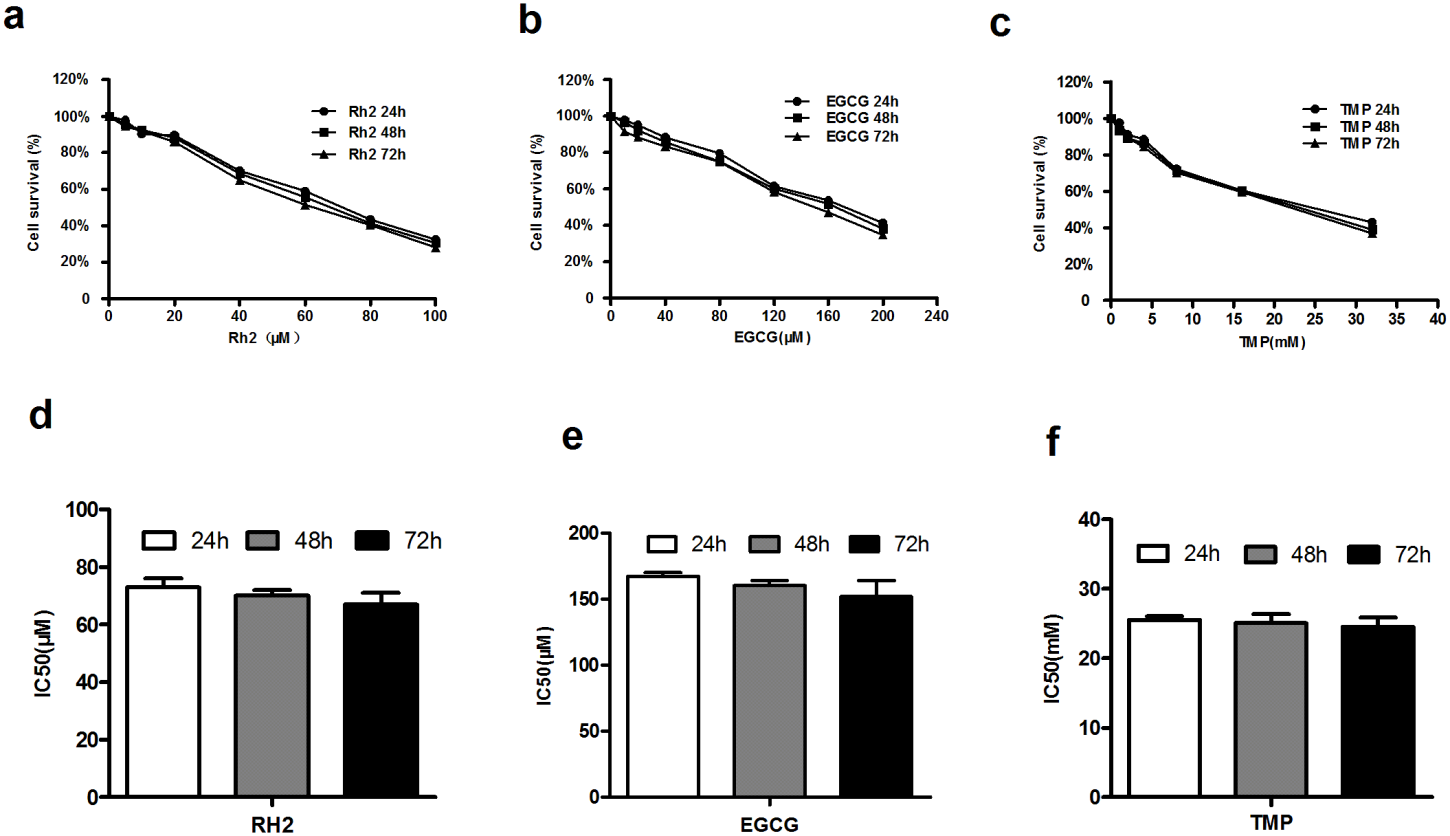
The inhibitory effect of three kind of herbal medicines in Pumc-91/ADM cell line. Cell survival curves (a,b,c) and IC_50_ values (d, e, f) of Rh2, EGCG and TMP for 24h, 48h and 72h, respectively.

Rh2, EGCG and TMP could inhibit the proliferation of Pumc-91/ADM with dose-dependency but no time-dependency (Fig 2, S1 Table). With the increase of Rh2, EGCG and TMP concentration, the growth of cells gradually decreased. After cells were treated with Rh2 (5–100 μM), EGCG (10–200 μM) and TMP (0–32 mM) for 24h, the increase of cell inhibition rate ranged from 2.1% to 58.7%, 2.2% to 67.7%, 2.4% to 56.9%, for 48h ranged from 3.3% to 61.9%, 4.4% to 69.4%, 6.4% to 60.9% and for 72h ranged from 8.5% to 65.2%, 5.5% to 71.9%, 6.6% to 62.9% in Pumc-91/ADM cells, respectively (Fig 2a, 2b and 2c). The inhibitory concentration 50% values (IC_50_) at 24h, 48h and 72h were estimated to be 73, 70, 67 μM for Rh2 (P>0.01) (Fig 2d), 167, 160, 152 μM for EGCG (P>0.01) (Fig 2e) and 25.51, 25.08, 24.00 mM for TMP (P>0.01) (Fig 2f), respectively.

### Reversal effects of Rh2, EGCG and TMP on multidrug resistance bladder cancer cells

**Fig 3:**
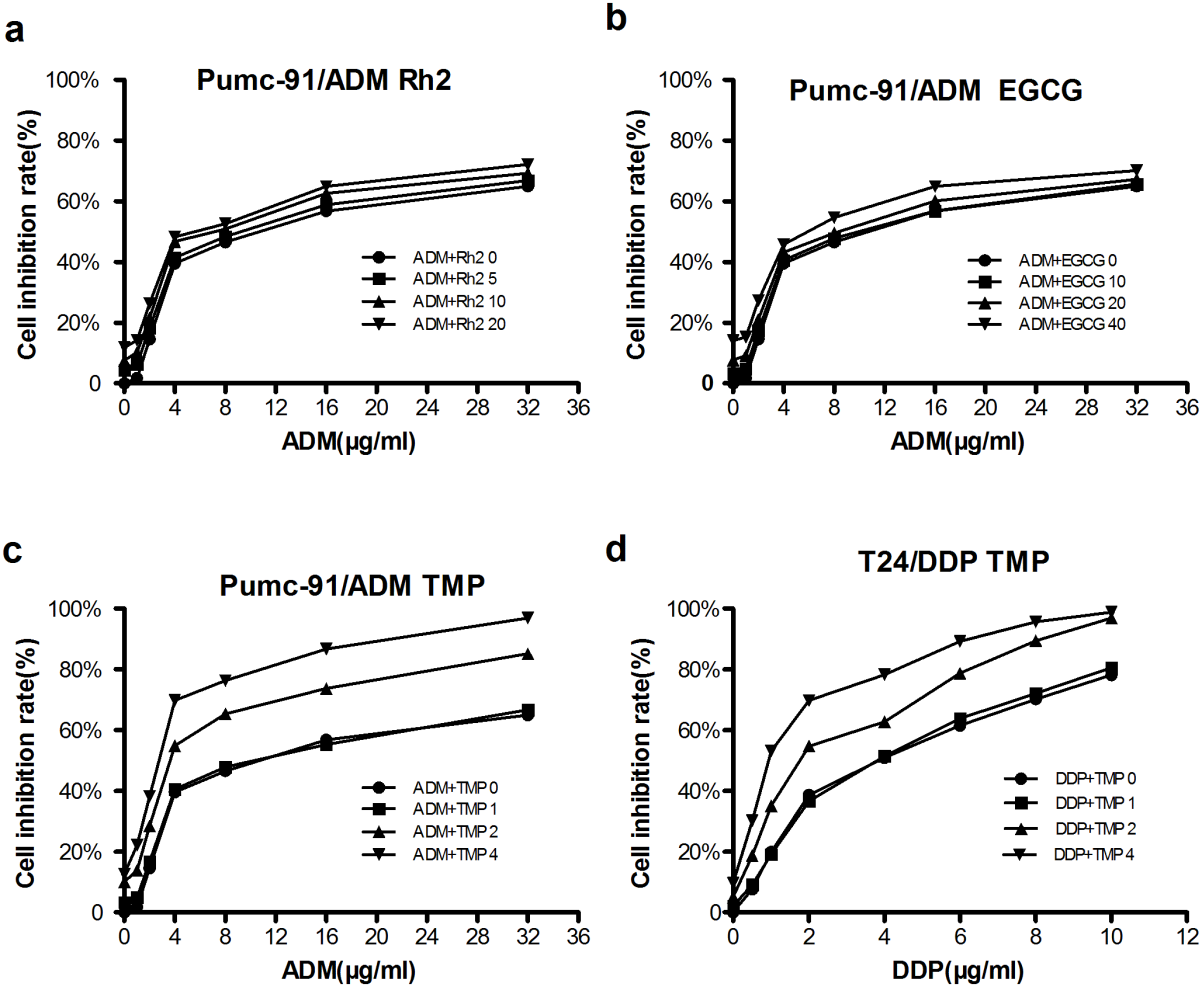
The reversal effects of three kind of herbal medicines on multidrug resistance bladder cancer cells. A range from 0 to 32 μg/ml of ADM was added into Pumc-91/ADM cells with (a) Rh2 (0, 5, 10, 20μM), (b) EGCG (0, 10, 20, 40μM) and (c) TMP (0, 1, 2, 4 mM) for 48 h. (d) A range from 0 to 10 μg/ml of DDP was added into T24/DDP cells with TMP (0, 1, 2, 4 mM).

We found that there was an obvious reversal effect of TMP among the three kinds of herbal medicines in Pumc-91/ADM cells (Fig 3c, S2 Table). But for Rh2 and EGCG (Fig 3a and 3b) (S2 Table), there were not. The IC_50_ values of ADM in combination with Rh2, EGCG or TMP for Pumc-91/ADM cells were shown in Table 1. Cells without herbal medicine were considered as the control group. Compared to the control group, the IC_50_ value of ADM in experimental groups treated with TMP was markedly decreased. The reversal fold was 1.05-fold (P>0.01), 2.48-fold (P<0.01) and 4.28-fold (P<0.01) in the groups of TMP at the concentration of 1mM, 2 mM and 4mM respectively. The combinational index (CI) was 0.99, 1.28 and 1.49 in TMP treated groups at the concentration of 1, 2 and 4 mM, respectively (Table 1). Meanwhile, the reversal effect of TMP on T24/DDP cells was also carried out (Fig 3d) (S3 Table). The reversal fold was 1.05-fold (P>0.01), 2.03-fold (P<0.01) and 3.25-fold (P<0.01) in the groups of TMP at the concentration of 1 mM, 2 mM and 4 mM, respectively. The combinational index (CI) was 0.98, 1.32 and 1.49 in TMP treated groups at the concentration of 1, 2 and 4 mM, respectively (Table 2). These results demonstrated that TMP had a reversal effect on multidrug resistance bladder cancer cells, with a dose-dependency.

**Table 1:** Effect of EGCG, Rh2 and TMP on ADM cytotoxicity in Pumc-91/ADM cells.

Group	IC_50_(μg/ml)	RF	CI
ADM	11.56	1.00	
ADM+Rh2 5	10.21	1.13	1.00
ADM+Rh2 10	8.40	1.38	1.05
ADM+Rh2 20	7.10	1.63	1.04
ADM+EGCG 10	10.94	1.06	0.98
ADM+EGCG 20	9.44	1.22	0.99
ADM+EGCG 40	7.55	1.55	1.00
ADM+TMP 1	11.04	1.05	0.99
ADM+TMP 2	4.67	2.48*	1.28
ADM+TMP 4	2.70	4.28*	1.49

ADM, adriamycin;TMP, tetramethylpyrazine; Rh2, ginsenoside; EGCG, (−)-Epigallocatechin gallate; IC_50_, half maximal inhibitory concentration; RF, reversal fold; CI, combine index.

* p<0.01.

**Table 2:** Effect of TMP on DDP cytotoxicity in T24/DDP cells.

Group	IC_50_(μg/ml)	RF	CI
DDP	3.57	1.00	
DDP+TMP 1	3.41	1.05	0.98
DDP+TMP 2	1.76	2.03*	1.32
DDP+TMP 4	1.10	3.25*	1.49

* p<0.01.

Nevertheless, RF was 1.13-fold, 1.38-fold, 1.63-fold (P>0.01) in the presence of Rh2 (5, 10 and 20 μM) and 1.06-fold, 1.22-fold, 1.55-fold (P>0.01) in the presence of EGCG (10, 20 and 40 μM), separately. The combinational index (CI) was 1.00, 1.05, 1.04 in Rh2 groups at the concentration of 5, 10, 20 μM and 0.98, 0.99, 1.00 in EGCG groups at the concentration of 10, 20, 40 μM, respectively (Table 1). These results indicated that Rh2 and EGCG both had no reversal effect on Pumc-91/ADM cells.

### Cell cycle arrested at S phase after treatment with TMP

**Fig 4:**
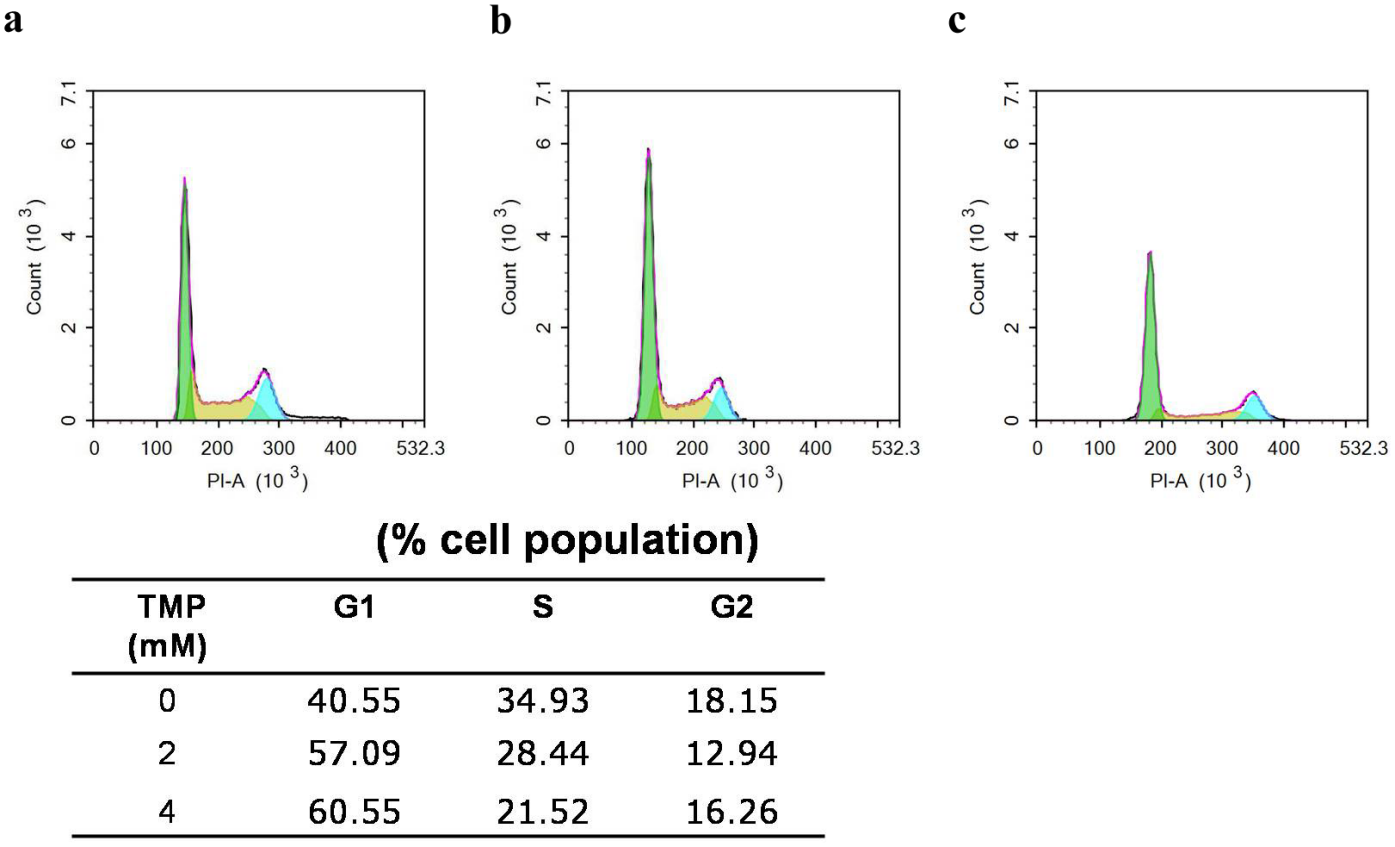
Cell cycle analysis of Pumc-91/ADM cells treated with TMP using flow cytometry. Cells were cultured with different concentrations of TMP (a. 0 mM, b.2 mM, c.4 mM) for 48 h. G1, S and G2 indicated cell cycle phases.

**Fig 5:**
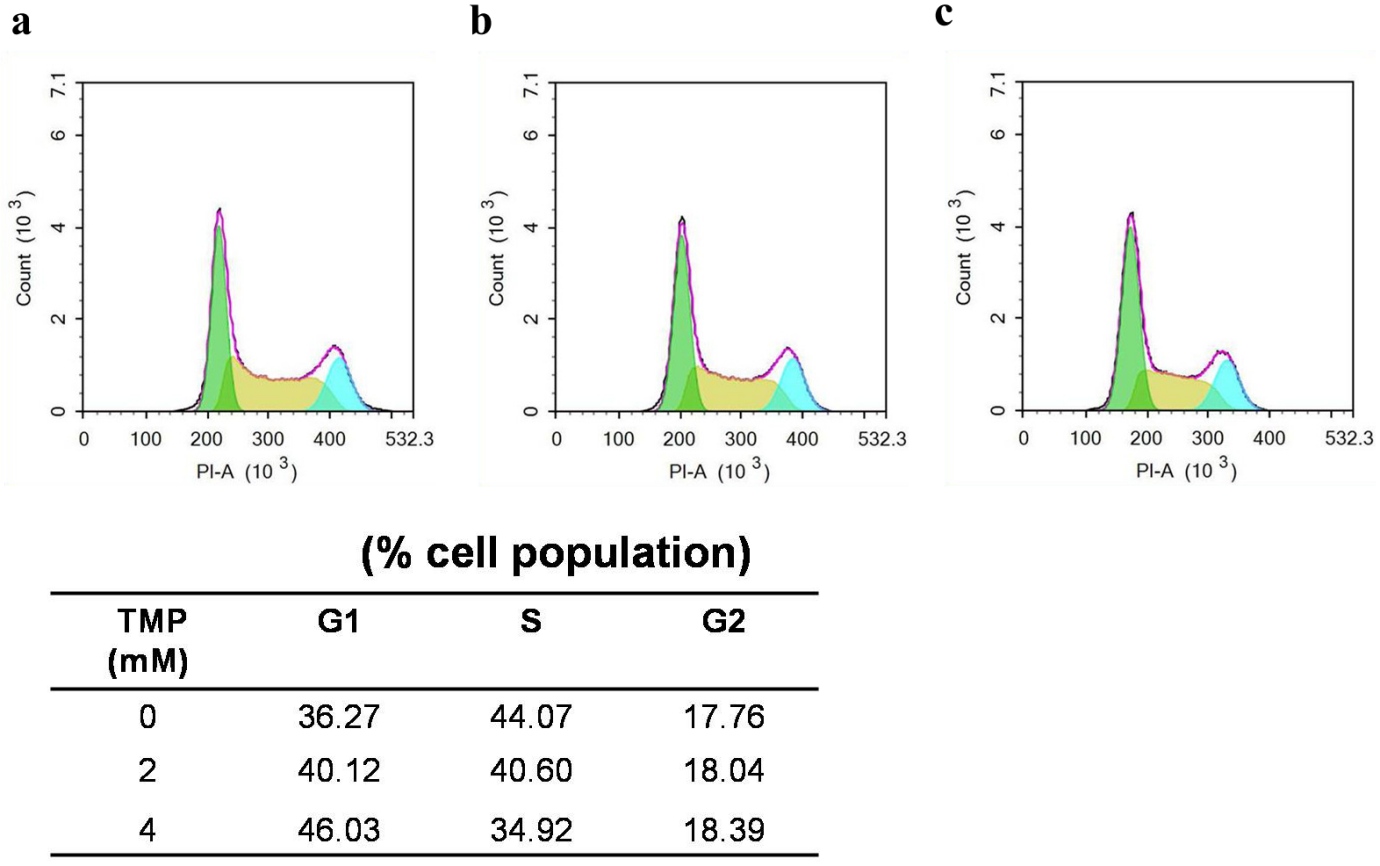
Cell cycle analysis of T24/DDP cells treated with TMP using flow cytometry. Cells were cultured with different concentrations of TMP (a. 0 mM, b.2 mM, c.4 mM) for 48 h. G1, S and G2 indicated cell cycle phases.

After Pumc-91/ADM and T24/DDP cells incubated with TMP for 48h, we examined the effect of TMP on cell cycle distributions. Compared to the control group, treatments with TMP (2, 4 mM) in experimental groups resulted in G1 phase arrest (57.09% and 60.55%, respectively, vs. 40.55%) in Pumc-91/ADM cells (Fig 4) and (40.12% and 46.03%, respectively, vs. 36.27%) in T24/DDP cells (Fig 5). These increases in the G1 cell population were accompanied with a decrease of S phase.

### Effects of TMP on mRNA levels of MRP1, LRP, GST, BCL-2 and TOPO-II in multidrug resistance bladder cancer cells

**Fig 6:**
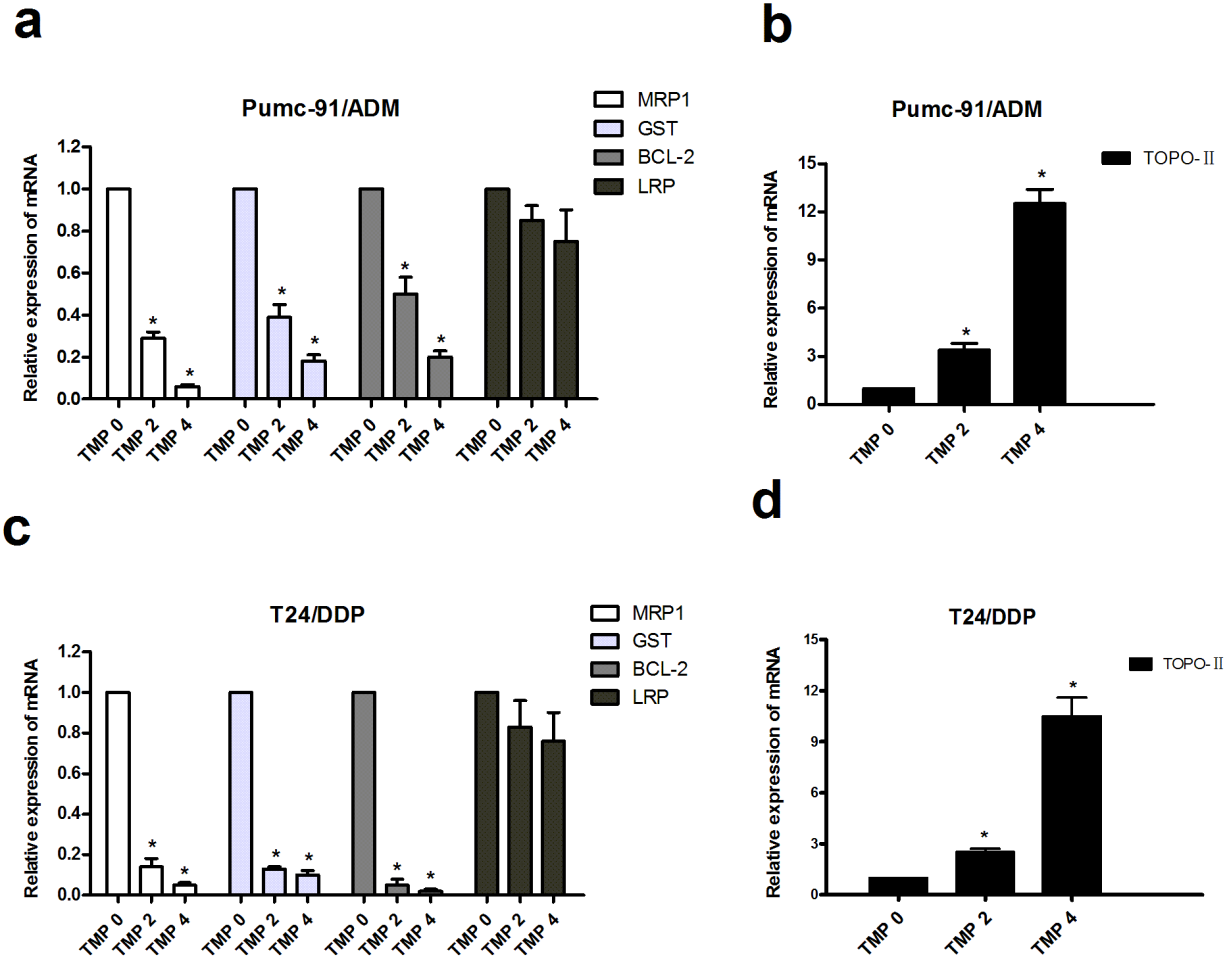
The relative expression of mRNA levels in Pumc-91/ADM and T24/DDP cells. Cells were treated with different concentrations of TMP (0, 2, 4 mM) for 48 h. The group in the absence of TMP was considered as the control group. Results were expressed as a ratio of target gene to housekeeping gene (GAPDH). *p<0.01 compared with the control group. a, b. The mRNA levels of MRP1, GST, BCL-2, LRP and TOPO-IIin Pumc-91/ADM cells. c, d. The mRNA levels of MRP1, GST, BCL-2, LRP and TOPO-II in T24/DDP cells.

To determine whether MRP1, LRP, GST, BCL-2 and TOPO-II mRNA expression are involved in the reversal effect of TMP on MDR, the levels of MRP1, LRP, GST, BCL-2 and TOPO-II in Pumc-91/ADM and T24/DDP cells treated with different concentrations of TMP were detected by qRT-PCR. TMP treatment caused decreases of MRP1, GST, BCL-2 (Fig 6a and 6c, S4 and S5 Tables) and an increase of TOPO-II (Fig 6b and 6d, S4 and S5 Tables) at the mRNA level in Pumc-91/ADM and T24/DDP cells. However, there was no difference in the protein of LRP after cells treated with TMP. As compared to the control group, the levels of mRNA in TMP groups at concentrations of 2, 4 mM were 0.29-fold, 0.06-fold lower for MRP1 (P<0.01), 0.39-fold, 0.18-fold lower for GST (P<0.01), 0.50-fold, 0.20-fold lower for BCL-2 (P<0.01) and 0.85-fold, 0.75-fold lower for LRP (P>0.01) in Pumc-91/ADM cells (Fig 6a), respectively. There were prominent decreases accompanied by a dose dependency in TMP treated groups. In T24/DDP cells, the levels of mRNA in TMP groups at concentrations of 2, 4 mM were 0.14-fold, 0.01-fold lower for MRP1 (P<0.01), 0.13-fold, 0.10-fold lower for GST (P<0.01), 0.01-fold, 0.02-fold lower for BCL-2 (P<0.01) and 0.83-fold, 0.76-fold lower for LRP (P>0.01) (Fig 6c). However, the expression of TOPO-II in TMP groups was increased. Compared to the control group, the levels of TOPO-II mRNA in TMP groups at concentrations of 2, 4 mM were 3.4-fold, 12.5-fold higher in Pumc-91/ADM cells and 2.5-fold, 10.5-fold higher at concentrations of 2, 4 mM in T24/DDP cells, respectively (Fig 6b and 6d).

### Effects of TMP on the protein levels of MRP1, LRP, GST, BCL-2 and TOPO-II in multidrug resistance bladder cancer cells

**Fig 7:**
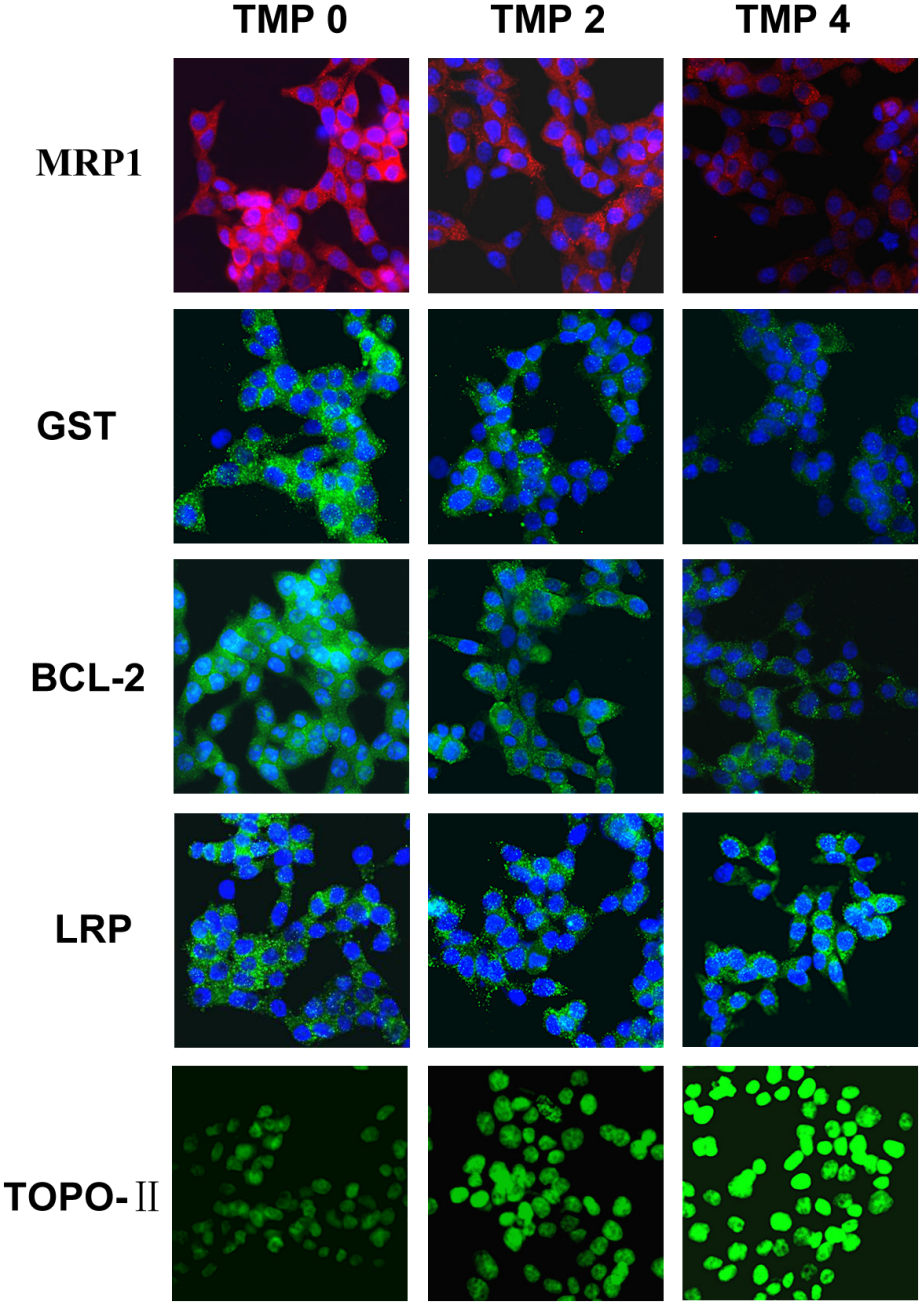
The expression of MRP1, LRP, GST, BCL-2 and TOPO-II at the protein levels examined by immunofluorescence assay in Pumc-91/ADM cells. Cells were treated with different concentrations of TMP (0, 2, 4 mM) for 48 h. The group in the absence of TMP was considered as the control group (magnification, ×400).

**Fig 8:**
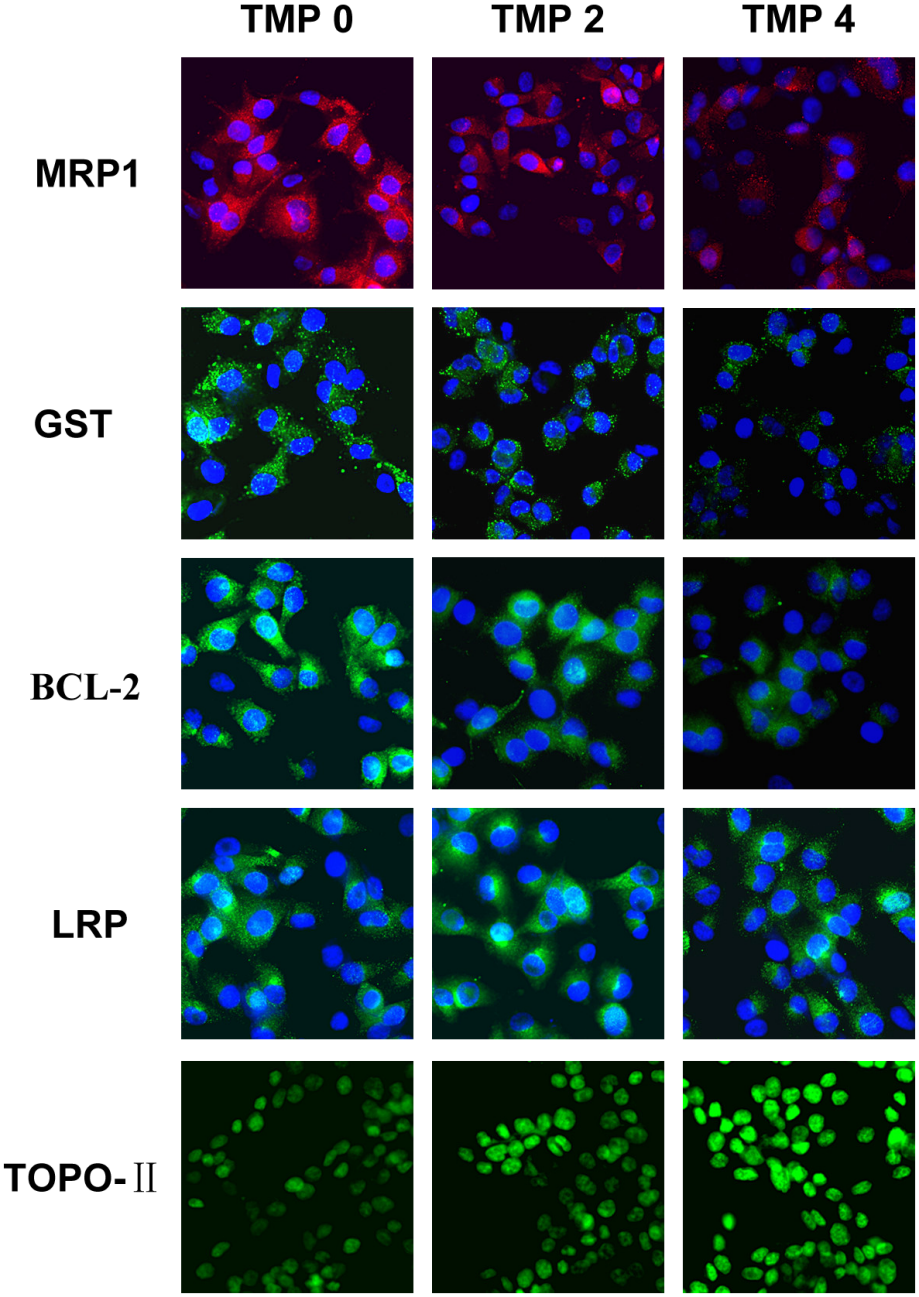
The expression of MRP1, LRP, GST, BCL-2 and TOPO-II at the protein levels examined by immunofluorescence assay in T24/DDP cells. Cells were treated with different concentrations of TMP (0, 2, 4 mM) for 48 h. The group in the absence of TMP was considered as the control group (magnification, ×400).

To investigate whether the mRNA levels of MRP1, LRP, GST, BCL-2 and TOPO-II in Pumc-91/ADM and T24/DDP cells were related to their protein expression of cells treated with TMP (2, 4 mM), immunefluorescence assay was carried out. Compared to the control group, the protein expressions of MRP1, GST, BCL-2 were significantly decreased in TMP groups (2, 4 mM) in accordance with their mRNA levels with a dose-dependency (Figs 7 and 8). But for TOPO-II, it was demonstrated that TMP could enhance its expression (Figs 7 and 8). With the concentrations of TMP increasing, the level of TOPO-II increased gradually in comparison to the untreated cells. But for LRP, there was no difference between the control group and TMP treated groups (2, 4 mM). These results indicated that TMP could down-regulate the expression of MRP1, GST, BCL-2 and up-regulate the expression of TOPO-II.

**Fig 9:**
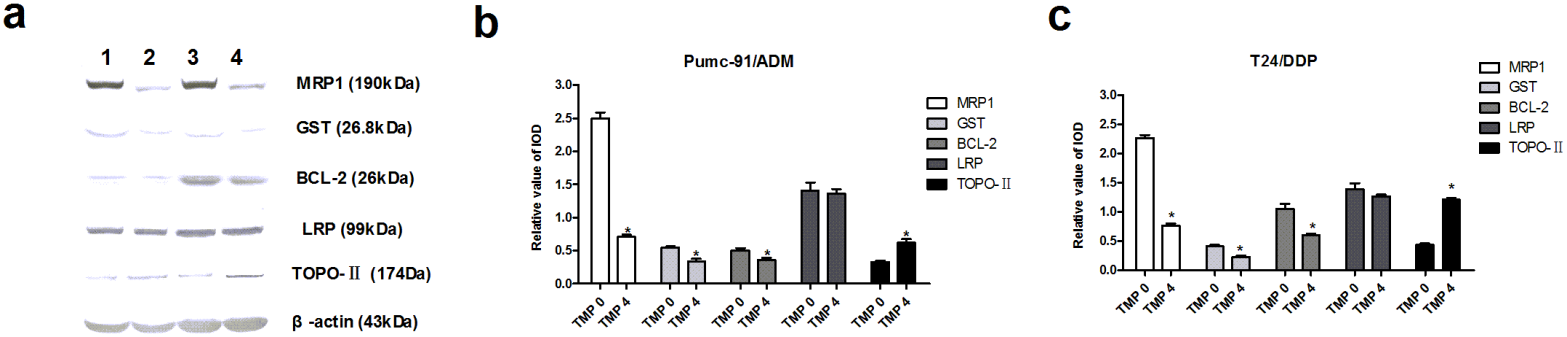
The expression of MRP1, GST, BCL-2, LRP and TOPO-II at the protein levels examined by western blot in Pumc-91/ADM and T24/DDP cells. a. electrophoregram. 1——Pumc-91/ADM TMP0, 2——Pumc-91/ADM TMP4, 3——T24/DDP TMP0, 4——T24/DDP TMP4. b, c. bar graph. *p<0.01 compared with the control group.

In order to quantify levels of these proteins, western blotting was performed on MRP1, GST, BCL-2, LRP and TOPO-II (Fig 9, S6 Table). The expression of MRP1, GST, BCL-2 was 0.28 fold, 0.62 fold and 0.72 fold lower in the TMP group than in the control group in Pumc-91/ADM cells (P<0.01), respectively. While the expression of MRP1, GST, BCL-2 was 0.34 fold, 0.56 fold and 0.57 fold lower in the TMP group than in the control group in T24/DDP cells (P<0.01). In contrast, TOPO-II expression was 1.9 fold and 2.75 fold higher in the TMP group compared to that in the control group in Pumc-91/ADM and T24/DDP cells, respectively (P<0.01). Whereas, there was no difference for LRP expression between the TMP groups and the control group in Pumc-91/ADM and T24/DDP cells.

## Discussion

Bladder cancer is one of the most important epithelial neoplasms worldwide. Patients with bladder cancer always show advanced stage disease in diagnosis, or relapse, invasion and dissemination after first-line therapy, causing a poor prognosis [26]. Adjuvant chemotherapy after surgery has been considered the major strategy for bladder cancer. Nevertheless, the main problem limiting the success of chemotherapy is the development of multidrug resistance [27,28]. Therefore, a combination therapy based on the synergistic effect between drugs has been used to overcome drug resistance. Herbal medicines are significant agents with pharmaceutical potential [29]. Modern pharmacological experiments have confirmed that the use of herbal medicines becomes increasingly popular, especially in cancer patients who exhibit chemotherapy resistance [30]. This approach may be a complementary and alternative therapy for chemotherapy. In consideration of the narrow therapeutic window of chemotherapy drugs, synergistic effect may improve therapeutic effects and reverse drug resistance because of the long-term chemotherapy.

Recently, many groups have reported synergistic anti-cancer effect in combination treatment with chemotherapeutic agents and a natural compound in vitro and in vivo cancer models [31,32]. It was reported that oral administration of Rh2 could inhibited the growth of ovarian xenografts in nude mice [33]. When Rh2 and cisplatin were intravenously administered to nude mice bearing ovarian xenografts, a synergistic effect was observed [34]. Synergy effect was also reported when murine hepatoma, sarcoma and melanoma models were treated with Rh2 and paclitaxel or mitoxantrone [35]. EGCG is synergistically cytotoxic to human cancer cells by modulating P-glycoprotein and the estrogen receptor [36,37]. It was also reported that EGCG could reverse cisplatin resistance through down regulating Axl and Tyro3 in human lung cancer cells [38]. TMP as one of the major bioactive components purified from the Chinese herb Ligusticum wallichii Franch., has been widely used in the treatment of cardiovascular and cerebral diseases in China [39]. It is reported that TMP acts as an anti-inflammatory agent in a rat asthma model [40]. Recently, many researchers have focused on the anti-tumorigenic activity in various cancer cells. Fu et al showed that TMP affected the growth and migration of the glioma cell line by the inhibition of calcium influx [41]. Similarly, it was confirmed that TMP could inhibit melanoma metastasis in vivo partly through suppressing the activity of vascular endothelial growth factor (VEGF) [42]. Therefore, TMP may constitute a potentially effective option for the treatment of tumors. One report demonstrated that TMP was capable of reversing the MDR of BEL-7402/ADM cells in response to ADM [21]. However, to the best of our knowledge, our research was the first time to detect the reversal effect of Rh2, EGCG and TMP in multidrug resistance bladder cancer cell line. In the present study, EGCG and Rh2 had no synergistic effect on bladder cancer. But for TMP, it was demonstrated that TMP was capable of enhancing the cytotoxicity of anticancer drugs by reversing MDR of Pumc-91/ADM cells in response to ADM. The reversal effect of TMP was also confirmed in T24/DDP cells. Compared to the control group, the IC_50_ of ADM and DDP was remarkably decreased with the concentration of TMP increasing, indicating that the reversal effect of TMP occurred with a dose-dependency. The exposure of Pumc-91/ADM and T24/DDP cells to TMP resulted in cell cycle arrest at G1 phase accompanied by a decrease of cell numbers in S phase. The important feature of cancer cells is that uncontrolled cell proliferation arises from defects throughout the cell cycle progression at G1, G2, S and mitotic phases. Cell cycle checkpoints, including a network of protein kinase signaling pathways, protect the cells from DNA damage induced by chemotherapeutics and provide the cells appropriate time to repair the damages [43]. Therefore, defects in cell cycle checkpoints could cause carcinogenesis and the development of drug resistance. Arrest of cell cycle progression at G2 phase generally gave the cells an opportunity to protect their viability after drug treatment [44,45]. In our study, cell cycle arrested at G1 phase inhibited uncontrolled cell proliferation and avoided the cell repairation for drug damage.

Drug resistance to chemotherapeutics occurs through several drug-metabolizing. Multi-drug resistance protein1 (MRP1) as an important ATP binding cassette transporter protein, affects the intracellular drug concentration through the alteration of drug influx or efflux. The overexpression of MRP1 can decrease intracellular concentration and cellular cytotoxicity of anticancer drug, such as adriamycin, vinblastine, mitoxantrone and etoposide [46–49]. GST has enzymatic activities with detoxification. It could decrease the concentrations of anticancer drugs through the GSH-conjugate export pump [50,51]. Cell death induced by apoptosis is one of the most common mechanisms of antineoplastic drugs. BCL-2 is considered as a main anti-apoptotic protein. In adriamycin-resistant H69AR human small cell lung cancer cells infected with BCL-2 interfering RNA, the level of BCL-2 decreased and also these cells were more sensitive to daunomycin than that of the parental cells [52]. Topoisomerase-II (TOPO-II) is the main target for many anticarcinogens. Drug resistance to TOPO-II occurs when the activity and sensitivity of the target enzyme TOPO-II are decreased by down-regulation or mutation [53]. Lung resistance protein (LRP) with a molecular weight of 99 kDa was identified as a major vault protein (MVP). It mediates drug resistance by pumping drugs away from intracellular drug targets through exocytotic vesicles or pump molecules [54]. Thus, we analyzed the expression of these drug resistance related proteins in this study. Our research demonstrated that there were obvious decreases in mRNA and protein levels of MRP1, GST, BCL-2 with TMP treatment in Pumc-91/ADM and T24/DDP cells. For TOPO-II, treatment with TMP resulted in an increase expression of TOPO-II in mRNA and protein levels. However, there was no difference in LRP expression between the control group and TMP treated groups. Thus, these dada demonstrated that TMP combined with adriamycin or cisplatin synergistically inhibited the proliferation of Pumc-91/ADM and T24/DDP cells. TMP could reverse the drug resistance of Pumc-91/ADM and T24/DDP cells.

Also, MDR expression and correlation with other receptors, such as Steroid and Xenobiotic Receptor (SXR) has been clearly demonstrated, as well as their correlation with clinical evolution and behavior. Jorge Rioja demonstrated that SXR expression had value as a predictor of survival independent of the standard pathological predictors for patients with invasive bladder cancer [55].

In conclusion, we demonstrated that TMP was able to enhance the cytotoxicity of anticarcinogen in Pumc-91/ADM and T24/DDP cells. Meanwhile, the mechanisms of this reversal effect were explored. TMP could induce accumulation of Pumc-91/ADM and T24/DDP cells at G1 phase in the cell cycle accompanied by a decrease of cell numbers in S phase. Several drug-metabolizing genes and proteins were detected after cells were treated with TMP. Compared to the control group, obvious decreases of MRP1, GST, BCL-2 and an increase of TOPO-II were shown in TMP treated groups at mRNA and protein levels. But for LRP, there was no difference between the control group and TMP groups. We concluded that MRP1, GST, BCL-2 and TOPO-II might be partly responsible for the reversal effects of TMP in Pumc-91/ADM and T24/DDP cells. These findings demonstrated that TMP might be a potential candidate for reversal effect of drug resistance in bladder cancer chemotherapy.

## Supporting Information

S1 Table The inhibitory effect of three kind of herbal medicines in Pumc-91/ADM cell line. Pumc-91/ADM cells were treated with Rh2, EGCG and TMP at 37°C for 24h, 48h and 72h, respectively.
(TIF)

S2 Table The reversal effects of three kind of herbal medicines on Pumc-91/ADM cells. A range from 0 to 32 μg/ml of ADM was added into Pumc-91/ADM cells with Rh2 (0, 5, 10, 20μM), EGCG (0, 10, 20, 40μM) and TMP (0, 1, 2, 4 mM) for 48 h.
(TIF)

S3 Table The reversal effects of TMP on T24/DDP cells. A range from 0 to 10 μg/ml of DDP was added into T24/DDP cells with TMP (0, 1, 2, 4 mM).
(TIF)

S4 Table The relative expression of MRP1, GST, BCL-2, LRP and TOPO-II at mRNA levels in Pumc-91/ADM cells. Cells were treated with different concentrations of TMP (0, 2, 4 mM) for 48 h. The group in the absence of TMP was considered as the control group.
(TIF)

S5 Table The relative expression of MRP1, GST, BCL-2, LRP and TOPO-II at mRNA levels in T24/DDP cells. Cells were treated with different concentrations of TMP (0, 2, 4 mM) for 48 h. The group in the absence of TMP was considered as the control group.
(TIF)

S6 Table The expression of MRP1, GST, BCL-2, LRP and TOPO-II at the protein levels examined by Western blot in Pumc-91/ADM and T24/DDP cells. Pumc-91/ADM and T24/DDP cells were treated with TMP at the concentration of 4 mM for 48h. Proteins levels were quantified by Image-Pro Plus v 6.0 software. A, Taget gene. B. β-actin.
(TIF)
