# Supplementary figures and images for: The Reversal Effect and Its Mechanisms of Tetramethylpyrazine on Multidrug Resistance in Human Bladder Cancer
Source: PLoS One. 2016 Jul 8;11(7):e0157759. doi: 10.1371/journal.pone.0157759 (PMC4938409; doi:10.1371/journal.pone.0157759)

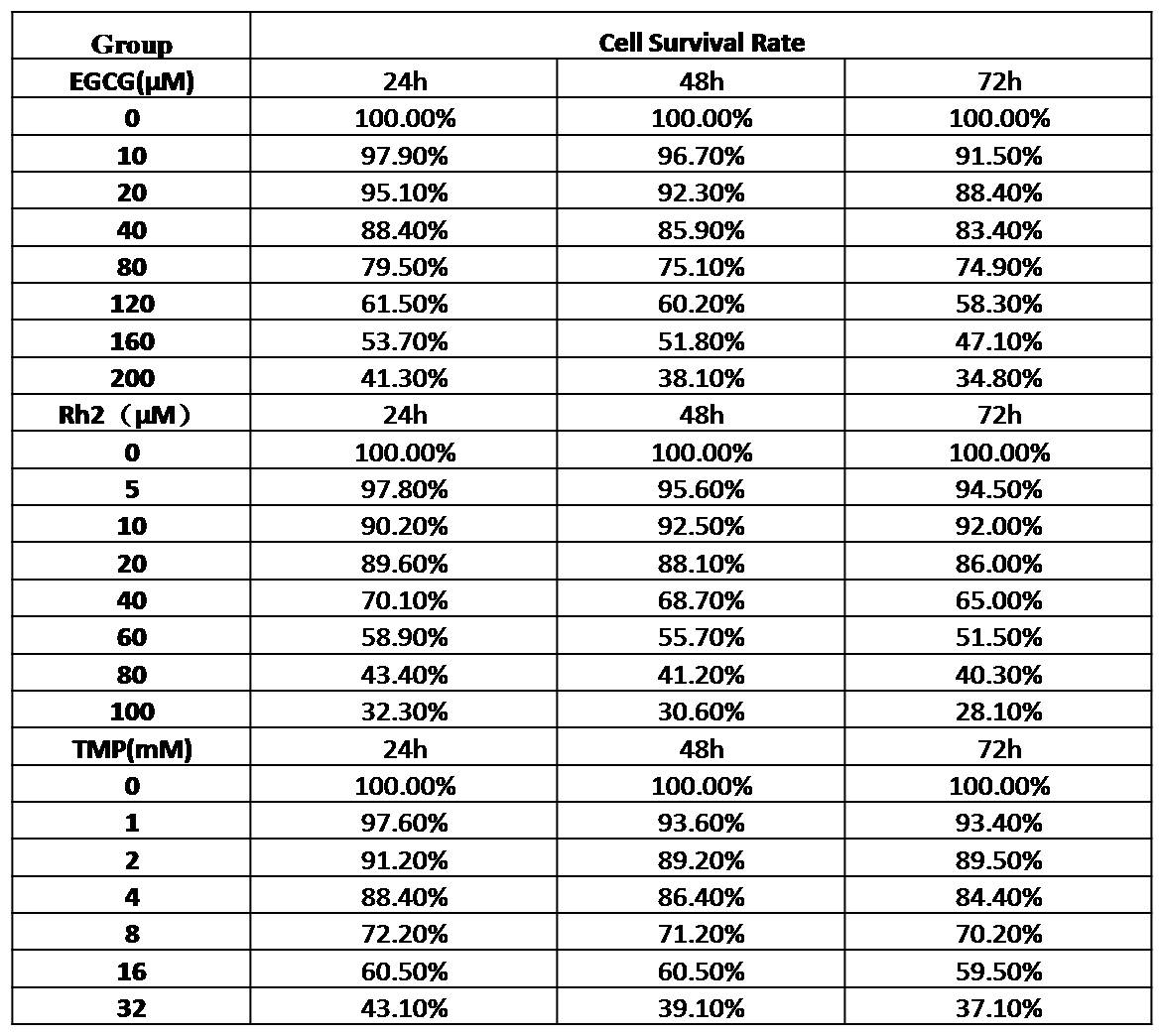

Supplement: S1 Table — Pumc-91/ADM cells were treated with Rh2, EGCG and TMP at 37°C for 24h, 48h and 72h, respectively. (TIF) [file pone.0157759.s001.tif]

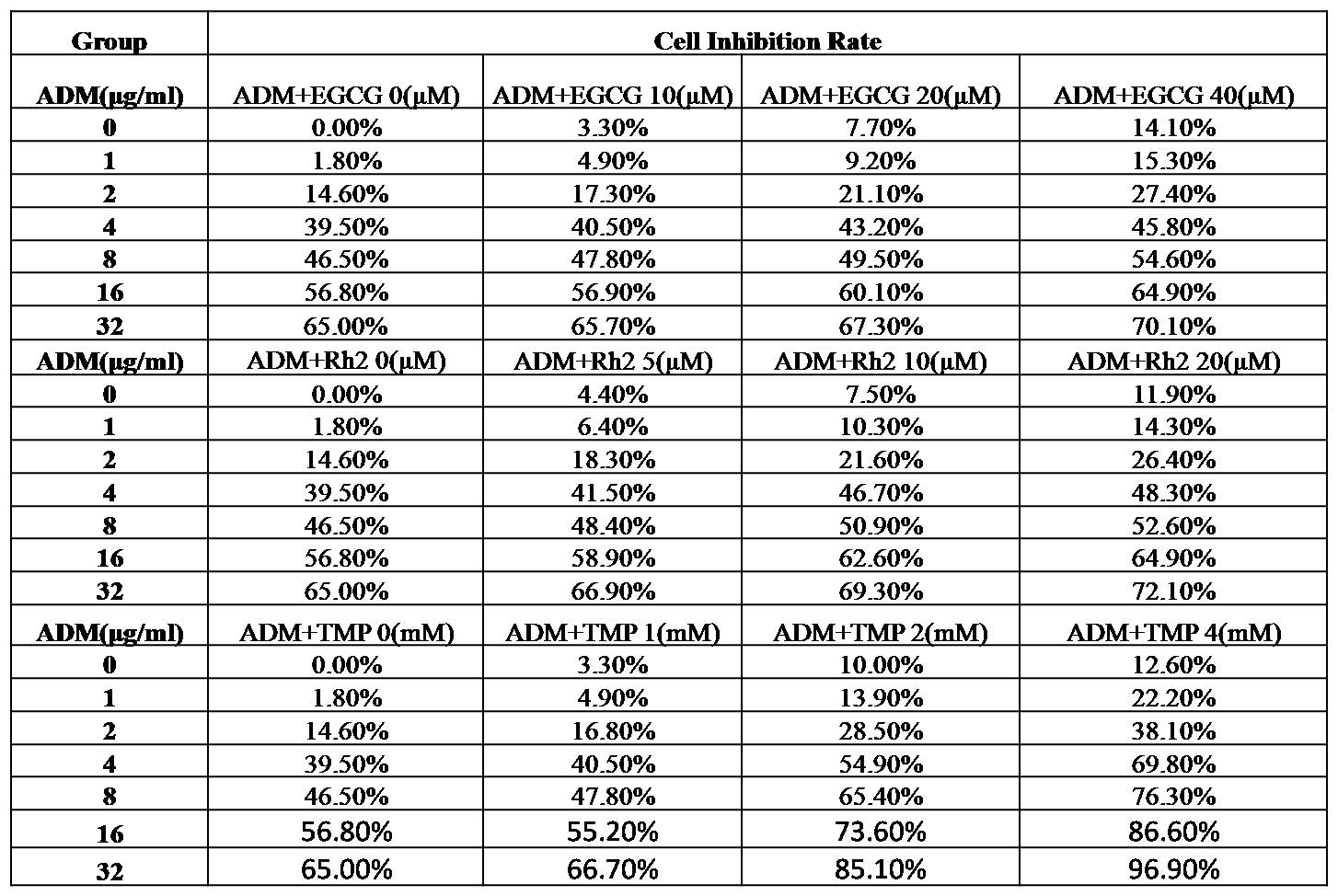

Supplement: S2 Table — A range from 0 to 32 μg/ml of ADM was added into Pumc-91/ADM cells with Rh2 (0, 5, 10, 20μM), EGCG (0, 10, 20, 40μM) and TMP (0, 1, 2, 4 mM) for 48 h. (TIF) [file pone.0157759.s002.tif]

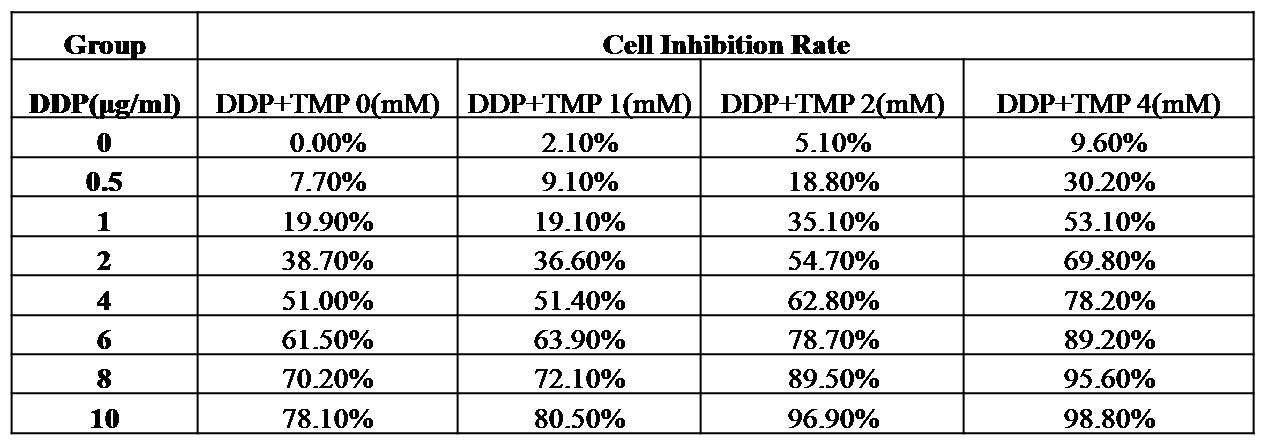

Supplement: S3 Table — A range from 0 to 10 μg/ml of DDP was added into T24/DDP cells with TMP (0, 1, 2, 4 mM). (TIF) [file pone.0157759.s003.tif]

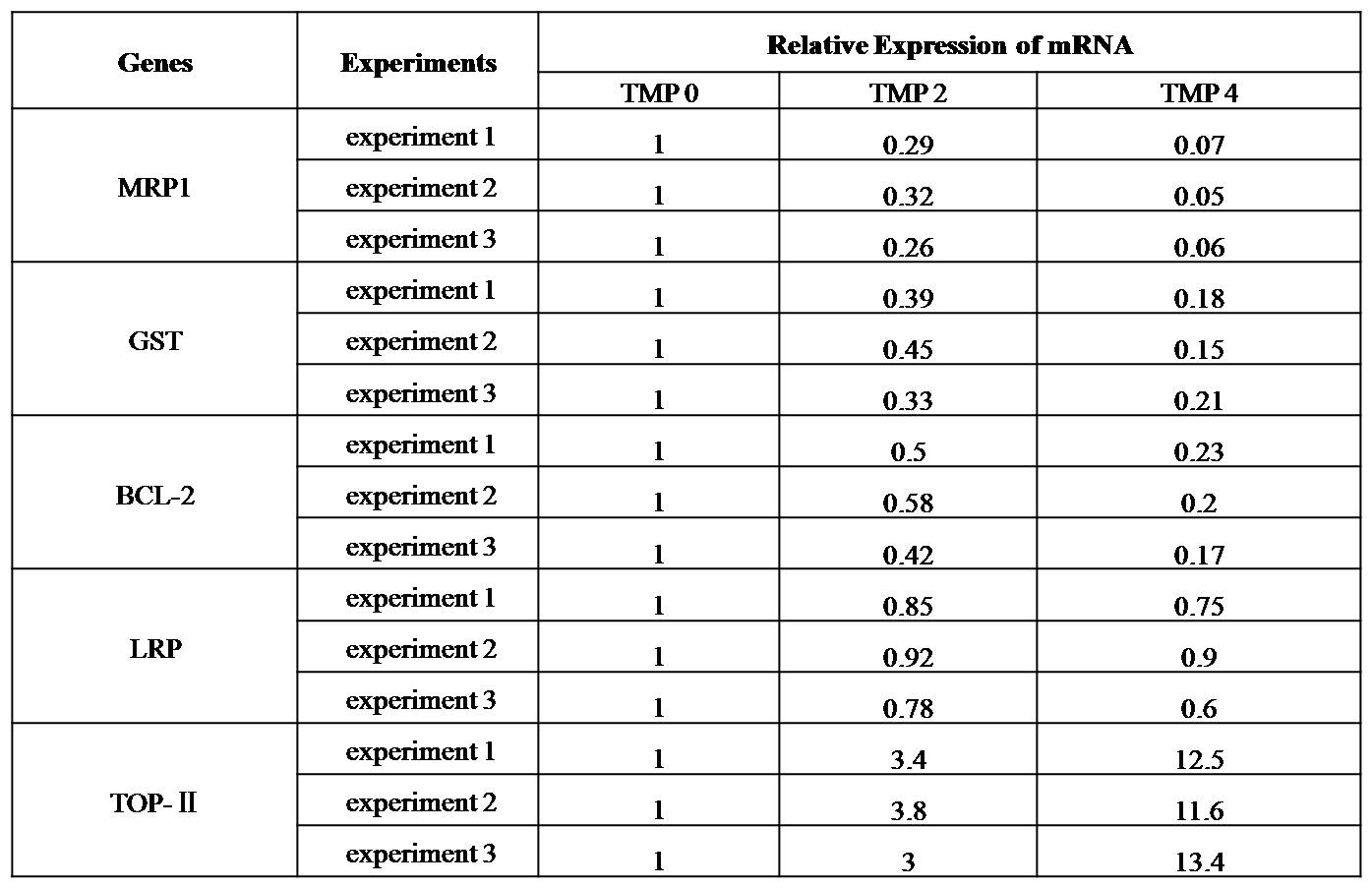

Supplement: S4 Table — Cells were treated with different concentrations of TMP (0, 2, 4 mM) for 48 h. The group in the absence of TMP was considered as the control group. (TIF) [file pone.0157759.s004.tif]

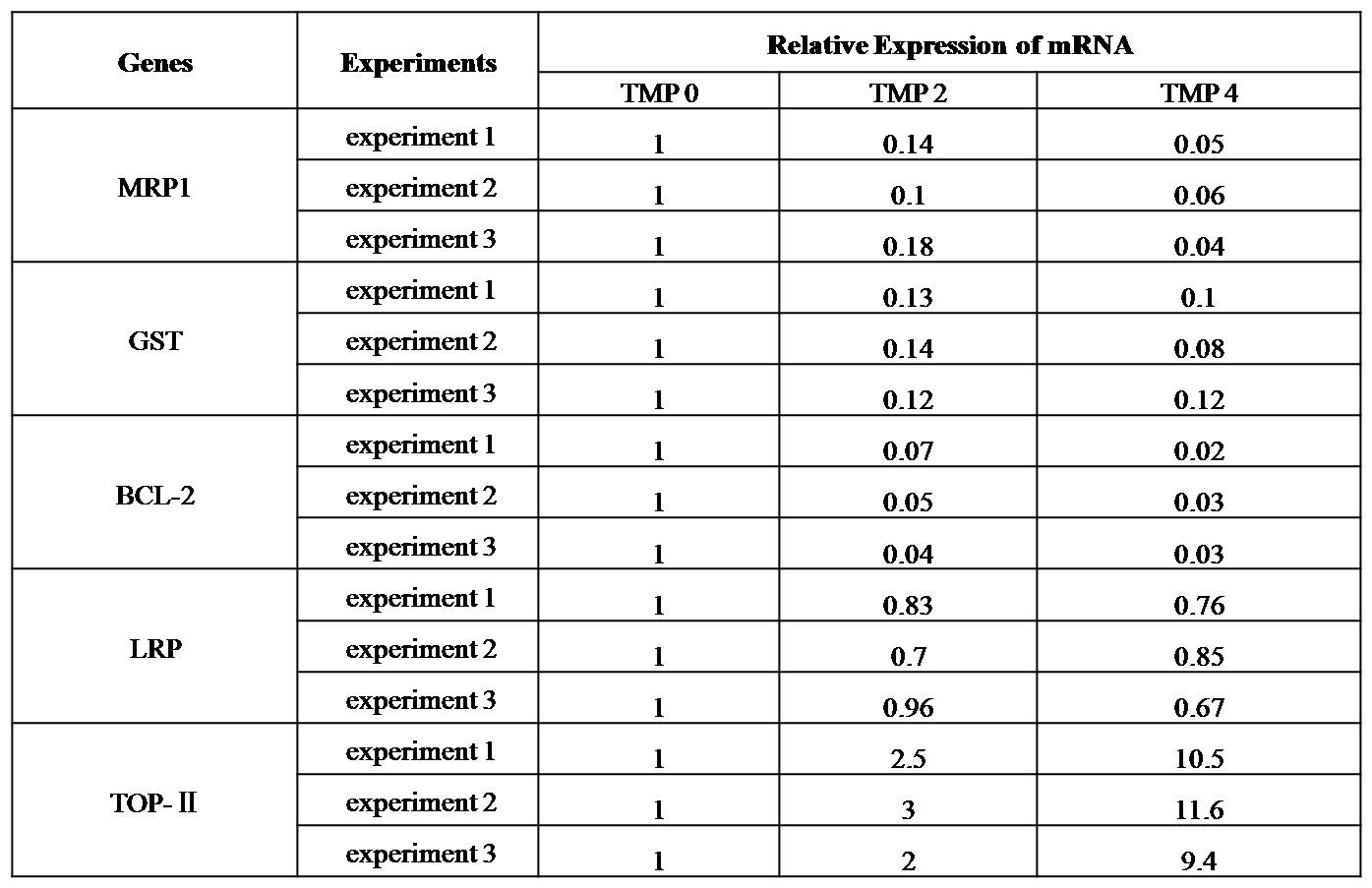

Supplement: S5 Table — Cells were treated with different concentrations of TMP (0, 2, 4 mM) for 48 h. The group in the absence of TMP was considered as the control group. (TIF) [file pone.0157759.s005.tif]

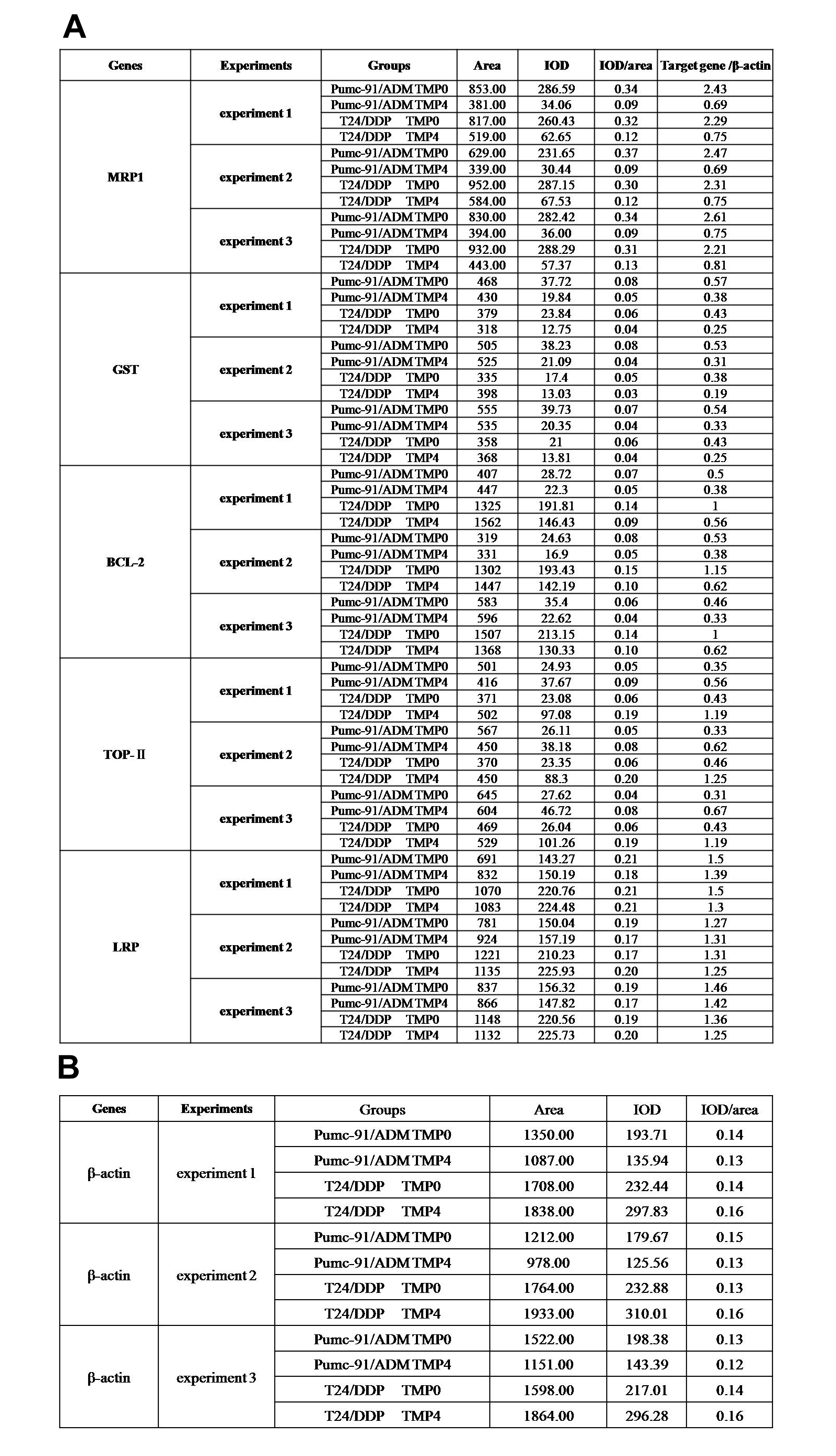

Supplement: S6 Table — Pumc-91/ADM and T24/DDP cells were treated with TMP at the concentration of 4 mM for 48h. Proteins levels were quantified by Image-Pro Plus v 6.0 software. A, Taget gene. B. β-actin. (TIF) [file pone.0157759.s006.tif]
